# The BET-bromodomain inhibitor JQ1 renders neuroblastoma cells more resistant to NK cell-mediated recognition and killing by downregulating ligands for NKG2D and DNAM-1 receptors

**DOI:** 10.18632/oncotarget.26736

**Published:** 2019-03-15

**Authors:** Irene Veneziani, Doriana Fruci, Mirco Compagnone, Vito Pistoia, Paolo Rossi, Loredana Cifaldi

**Affiliations:** ^1^ Department of Immunology, Bambino Gesù Children's Hospital, IRCCS, Rome, Italy; ^2^ Department of Pediatric Hematology and Oncology, Bambino Gesù Children's Hospital, IRCCS, Rome, Italy; ^3^ Academic Department of Pediatrics (DPUO), Bambino Gesù Children's Hospital, Rome, Italy; ^4^ Department of Systems Medicine, University of Rome Tor Vergata, Rome, Italy

**Keywords:** neuroblastoma, MYCN oncogene, BET-bromodomain inhibitor JQ1, ligands for NK cell-activating receptors, tumor immune escape

## Abstract

Low expression of ligands for NK cell-activating receptors contributes to neuroblastoma (NB) aggressiveness. Recently, we demonstrated that the expression of MYCN, a poor prognosis marker in NB, inversely correlates with that of activating ligands. This indicates that MYCN expression level can predict the susceptibility of NB cells to NK cell-mediated immunotherapy and that its downregulation can be exploited as a novel therapeutic strategy to induce the expression of activating ligands. Here we evaluated the effect of the BET-bromodomain inhibitor JQ1 on the expression of ligands for NK cell-activating receptors in NB cell lines. Although downmodulating MYCN, JQ1 impaired the expression of ligands for NK cell-activating receptors, rendering NB cell lines more resistant to NK cell-mediated killing. The downregulation of activating ligands was due to JQ1-mediated impaired functions of both c-MYC and p53, two transcription factors known to regulate the expression of ULBP1-3 ligands for NKG2D activating receptor. Moreover JQ1 strongly downregulated the levels of ROS, a stress-induced signaling event associated with the induction of ligands for NK cell-activating receptors. These results suggest that the use of JQ1 should be discourage in combination with NK cell-based immunotherapy in a perspective chemotherapeutic treatment of NB. Thus, further investigations, exploiting molecular strategies aimed to boost the NK cell-mediated killing of NB cells, are warranted.

## INTRODUCTION

Neuroblastoma (NB), the extracranial tumor arising from the aberrant persistence of neural crest progenitors during sympathetic nervous system development, accounts for 15% of all pediatric cancer deaths [1]. Among several chromosomal aberrations occurring during the embryonal life in neuroectodermal cells, causing NB tumorigenesis, the amplification of *MYCN* oncogene is the best established marker of poor prognosis.

Cancer cells, including NB, can subvert both adaptive and innate antitumor immune responses through several mechanisms [2, 3], including downregulation of ligands for NK cell-activating receptors, thus contributing to tumor progression and relapse [4, 5].

NK cells are cytotoxic lymphocytes belonging to the innate immune system involved in the control of viral infected and transformed cells without prior specific sensitization [6, 7]. Their function is regulated by the tuned activity of both activating and inhibitory receptors binding to specific ligands expressed on the surface of target cells. In particular, NK cell-mediated recognition and lysis of cancer cells is dependent on the expression of ligands for NKG2D and DNAM-1 NK cell-activating receptors on tumor cells [8]. The ligands for these two receptors (MICA, MICB and ULBP1-6 for NKG2D receptor and PVR/CD155 and Nectin2/CD122 for DNAM-1 receptor) are expressed on different type of tumor cells and induced by several anticancer drugs [9].

The mechanisms regulating the expression of ligands for these NK cell-activating receptors are still partially understood. *ULBP1*, *ULBP2* and *ULBP3* genes are regulated by c-MYC and p53 transcription factors [10, 11]. As known, the *p53* gene is rarely mutated in NB at diagnosis [12]. P53 function is regulated by a complex network of molecules, including MDM2 [13, 14]. Of note, both p53 and MDM2 are direct MYCN transcriptional targets and consequently co-expressed at high levels in *MYCN*-amplified NB cells [15, 16]. Accordingly, p53 function is suppressed by MDM2 in *MYCN*-amplified NB cells. Moreover, in NB the expression of MYCN inversely correlates with that of c-MYC [17, 18]. Furthermore, the expression levels of ligands for NK cell-activating receptors have been associated with those of reactive oxygen species (ROS), generally produced by stress-induced signaling events [19–21].

We asked whether NB aggressiveness, associated with *MYCN* amplification, could be related to mechanisms of immune escape involving downregulation of ligands for NK cell-activating receptors. Recently, we demonstrated that the expression of MYCN is inversely correlated with that of ligands recognized by NKG2D- and DNAM-1-activating receptors in both human NB cell lines and NB patient specimens [18]. Downregulation of MYCN, by using the conditionally MYCN-expressing Tet-21/N cell line, results in enhanced expression of ligands for NKG2D and DNAM-1 NK cell receptors by rendering NB cells more susceptible to NK cell-mediated recognition and killing. These data reveal that *MYCN* overexpression protects NB cells from NK cell-mediated anti-tumor activities, thus delineating a novel mechanism of tumor immune-escape based on the repression of ligands for NK cell-activating receptors. The expression of MYCN could therefore represent a biomarker to predict the susceptibility of NB cells to NK cell-mediated immunotherapy [18].

In view of these data [18], we explored molecular strategies aimed to inhibit MYCN functions in order to enhance the expression of ligands for NK cell-activating receptors in NB. In general, MYCN drives NB tumorigenesis through the induction of several target genes involved in many pathways regulating tumor cell proliferation, growth, apoptosis, energy metabolism, and differentiation [22, 23]. In normal conditions, MYCN is expressed during the embryogenesis in several tissues and is downregulated after the embryonic development reaching not significant levels in adult tissues [23]. MYCN plays an important role in the development of normal brain [24]. By opposite, in malignancies including NB, aberrant amplification and/or overexpression of MYCN have been associated with tumor aggressiveness with MYCN-amplified cells having stem like characteristics and a pluripotent state [25]. Since several evidences suggest a causal role of MYCN in the development of NB and in other tumor types, while its expression is negative in normal tissues, MYCN oncogene may represent an attractive cancer therapeutic target. However, the downregulation of MYCN is still very challenging. Among several approaches used, currently the BET-bromodomain inhibitor JQ1 represents a good candidate, impairing cell growth and inducing apoptosis [26]. JQ1, targeting BRD4 [27], efficiently downregulates the expression of both MYCN and c-MYC [28]. This small-molecule has been extensively shown to exert different anti-tumor activities in several malignancies, including NB [29], by inducing DNA damage response, growth arrest and apoptosis [30, 31], inhibiting angiogenesis [32] and reducing hypoxia [33]. BET-bromodomain inhibitors are used for treatment of several types of cancer, as reported in https://www.clinicaltrials.gov/ website. Of note, JQ1 promotes the anti-tumor immunity by reducing the expression of programmed death ligand 1 (PD-L1) [34], an immune checkpoint molecule expressed in NB microenvironment [35]. Thus, to assess the immunomodulatory effect of JQ1, we treated p53 wild-type NB cell lines with JQ1 and, at pre-apoptotic dose, we evaluated the expression levels of activating ligands and NB cell susceptibility to NK cell-mediated recognition and lysis in response to JQ1-mediated MYCN downregulation. Our data showed that JQ1 impaired the function of both c-MYC and p53 transcription factors and strongly reduced the level of reactive oxygen species (ROS), thus affecting the expression levels of ligands for NK cell-activating receptors and, consequently, rendering NB cells more resistant to NK cell killing. This impaired immunomodulation effect suggests that the use of JQ1 should not be combined with the prospective and promising NK cell-based immunotherapy of NB.

## RESULTS

### JQ1 affects the expression of ligands for NK cell-activating receptors in NB cell lines

**Figure 1:**
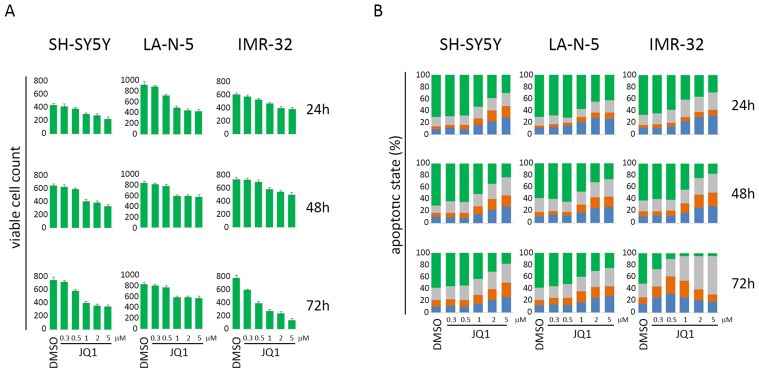
Cell count and apoptotic state of NB cell lines upon JQ1 treatment SH-SY5Y, LA-N-5 and IMR-32 NB cell lines were treated with DMSO or JQ1 at the indicated concentrations for 24, 48 or 72 hours. **(A)** Viable cell count was performed at each condition by trypan blue exclusion-based method. **(B)** Apoptotic state was evaluated by Annexin V and propidium iodide (PI) staining and flow cytometry analysis. The percentage of viable cells (Annexin V-negative PI-negative, green bar), cells in early phase of apoptosis (AnnexinV-positive PI-negative, blue bar), cells in late phase of apoptosis (Annexin V-positive PI-positive, orange bar) and dead cells (Annexin V-negative PI-positive, grey bar) are indicated in stacked histograms. A representative experiment out of three performed is shown.

**Figure 2:**
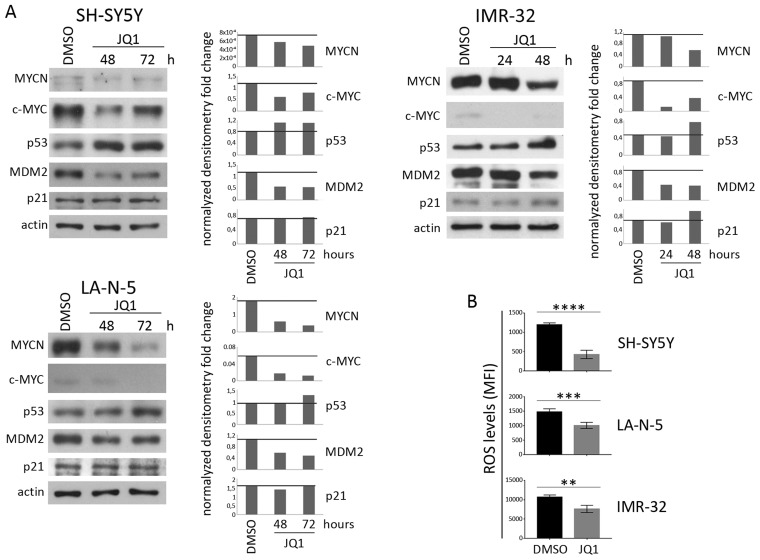
Expression levels of c-MYC, p53 and ROS production in JQ1-treated NB cell lines NB cell lines SH-SY5Y, LA-N-5 and IMR-32 were either treated with DMSO as control or with a pre-apoptotic dose of JQ1 (0.5 μM) for the time indicated. **(A)** Representative example out of three independent experiments of immunoblot analysis of MYCN, c-MYC, p53, MDM2 and p21 (left panel of each cell line). An anti-actin Ab was used for normalization. Densitometry analysis of actin-normalized protein values are shown (right panel of each cell line). **(B)** NB cell lines were treated with DMSO or 0.5 μM of JQ1 for 48 hours and ROS production was measured by flow cytometry. ROS levels were expressed as mean ± SD of mean fluorescence intensity (MFI) obtained by four independent stainings for each NB cell line (two-tailed unpaired Student's *t*-test); ^**^p<0.01, ^***^p<0.001, ^****^p<0.0001.

The *MYCN* non-amplified (non-MNA) SH-SY5Y and *MYCN*-amplified (MNA) LA-N-5 and IMR-32 NB cell lines were cultured in the presence of JQ1 at different doses for 24, 48 and 72 hours in order to identify optimal conditions enabling cell viability and preventing apoptosis which could compromise the surface expression of ligands. Cell viability and apoptosis were assessed by cell count and AnnexinV/PI staining, respectively. The dose of 0.5 μM did not affect cell growth (Figure 1A) or apoptotic state (Figure 1B) compared with higher drug doses at all three time points tested. At 72 hours, IMR-32 cell line was more sensible to JQ1 treatment compared with the others NB cell lines, as evaluated by cell growth arrest and apoptosis induction even at low JQ1 doses (Figure 1A and 1B). Then, we performed Western blotting analysis to evaluate the effect of JQ1 on the expression level of MYCN, together with that of c-MYC, p53 and the two main p53 functional readouts, MDM2 and p21. As shown in Figure 2A, JQ1 treatment (0.5 μM for 48 or 72 hours for both SH-SY5Y and LA-N-5, and 0.5 μM for 24 or 48 hours for IMR-32) efficiently downregulated MYCN in all NB cell lines, with more evident effects on both MNA LA-N-5 and IMR-32 cell lines. JQ1 downregulated also c-MYC as evident in SH-SY5Y, consistent with the fact that the promoter of *c-MYC* shares BET-bromodomains with that of *MYCN* oncogene [28]. These data indicate that in NB cell lines JQ1 impaired the expression of c-MYC, one of the two transcription factors known to regulate the expression of ligands for NK cell-activating receptors [10]. Moreover, p53 protein levels were very weakly increased upon JQ1 treatment with a significant decrease of MDM2. As p53 functional readout, we also evaluated p21 that, upon JQ1 treatment, did not change in both SH-SY5Y and LA-N-5 and very weakly increased in IMR-32. We supposed that the weak increase of p53 levels results from a double mechanism: on one side the down-regulation of MYCN that should down-regulate p53 levels, and on the other side the reduction of MDM2 that leads to a partial p53 stabilization which, however, is not functional enough as evaluated by the unmodified p21 level. A slight up-modulation of p53 was previously observed also in conditionally MYCN-expressing Tet-21/N cell line at early time points (8-16 hours of Doxycycline treatment) followed by a reduction at later time points (24-72 hours of Doxycycline treatment), suggesting a transient p53 stabilization upon MYCN down-modulation [18]. The high toxicity of JQ1 did not provide us to evaluate a potential down-regulation of p53 at later time points by Western blotting analysis. These data indicate that JQ1 did not restore p53 function, the second transcription factor known to regulate the expression of activating ligands [11].

Next, we evaluated JQ1-treated NB cell lines for the production of ROS that has been extensively reported to be correlated with the expression of ligands for NK cell-activating receptors [19–21]. As shown in Figure 2B, 0.5 μM of JQ1 at 48 hours strongly down-regulated the levels of ROS in all three NB cell lines. These data are in line with other evidences, in different tumor types, showing that JQ1 treatment is able to impair several BRD4 genes involved in the production of ROS [36, 37]. Overall, these data indicate that a pre-apoptotic dose of JQ1 in NB cell lines, in addition to inhibiting the expression of c-MYC and keeping impaired p53 function, it additionally alters a stress-induced event as ROS production, which is also involved in the induction of activating ligands.

**Figure 3:**
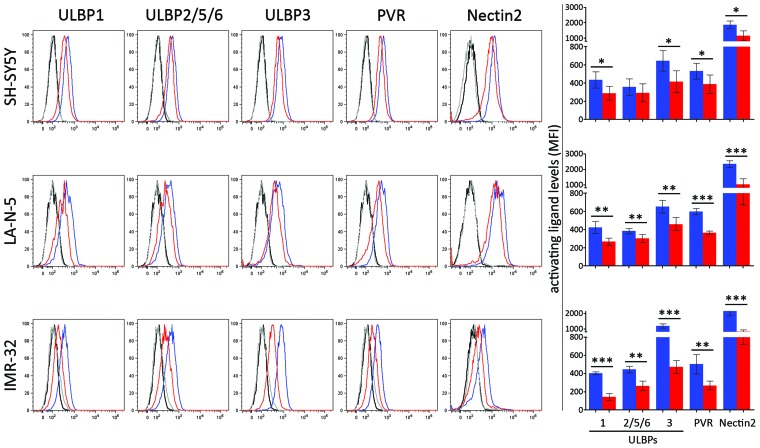
JQ1 downregulates the expression of ligands for NK cell-activating receptors NB cell lines SH-SY5Y, LA-N-5 and IMR-32 were treated with DMSO (blue bar) or JQ1 (0.5 μM, red bar) for 48 hours and surface expression of ligands for NK cell-activating receptors was evaluated by flow cytometry analysis. Isotype-matched negative control antibodies are displayed as black and grey lines for DMSO- and JQ1-treated NB cell lines, respectively. Representative flow cytometry analysis of surface expression of each ligands for NK cell-activating receptors (right panel) and the summary expressed as mean ± SD of MFI of five independent experiments (left panel) were reported. *p* values, compared DMSO- and JQ1-treated NB cell lines (two-tailed unpaired Student's *t*-test); ^*^p<0.05, ^**^p<0.01, ^***^p<0.001.

The effect of JQ1-mediated MYCN downregulation was evaluated on the surface expression of activating ligands by flow cytometry analysis. The expression levels of ULBP1, ULBP3, PVR and Nectin-2 were significantly downregulated in SH-SY5Y, LA-N-5 and IMR-32 cell lines upon JQ1 treatment (Figure 3). These data indicate that in spite of an efficient MYCN downregulation mediated by JQ1, the impaired function of both c-MYC and p53 and the reduced levels of ROS led to a compromised expression of ligands for NK cell-activating receptors.

These results were consistent with our previous data regarding the modulation of MYCN through a Tet-off cellular model in which we appreciated the increase of activating ligands only in conditions of up-modulation of c-MYC and p53 [18]. Here, these evidences confirmed that the molecular strategies aimed to downregulate MYCN in order to treat NB should be accomplished by a sustained activation of all the events, excluding those inducing side effects (such as c-MYC up-modulation), involved in the induction of ligands for NK cell-activating receptors.

### JQ1-treated NB cell lines are less susceptible to NK cell-mediated recognition and killing

**Figure 4:**
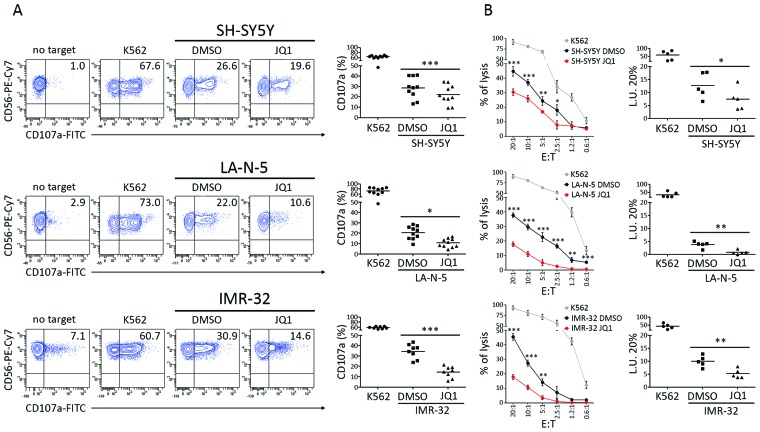
JQ1 renders NB cell lines more resistant to NK cell-mediated recognition and lysis DMSO and JQ1-treated SH-SY5Y, LA-N-5 and IMR-32 cell lines were used as target cells in NK-cell functional assays. **(A)** Degranulation of human CD3^-^CD56^+^CD45^+^ NK cells isolated from healthy donors, measured as cell surface expression of CD107a marker, following stimulation with SH-SY5Y, LA-N-5 and IMR-32, either treated with DMSO or JQ1 (0.5 μM) for 48 hours. K562 cell line was used as positive control. The percentage of CD107a^+^ NK cells is indicated. A representative experiment out of ten performed is shown (left panel). Summary of NK cell-degranulation of cells isolated from ten healthy donors is shown (right panel). Dots, percentage of CD107a^+^ NK cells; horizontal bars, average values. **(B)** DMSO- and JQ1-treated SH-SY5Y, LA-N-5 and IMR-32 cell lines were used as targets for NK cells isolated from five healthy donors in a standard ^51^Cr-release assay at the indicated effector:target (E:T) ratios. One representative experiment out of the five performed is shown (right panel). Specific lysis was converted to L.U. 20%. Summary of cytotoxic assays of NK cells isolated from five healthy donors are reported (left panel). Dots, L.U. 20% of the effector/target pairs tested; horizontal bars, average values. In A and B, *p* value, compared with DMSO- and JQ1-treated NB cell lines (two-tailed unpaired Student's *t*-test); ^*^p<0.05, ^**^p<0.01, ^***^p<0.001.

To evaluate if the decreased expression levels of activating ligands could affect NK cell-mediated recognition and killing of NB cells, we performed both NK cell degranulation and ^51^chromium release assay using DMSO- and JQ1-treated NB cell lines as targets. Consistently with the decreased levels of activating ligands, JQ1-treated NB cell lines were significantly more resistant to NK cell-mediated degranulation and cytotoxicity (Figure 4A-4B).

These data suggested that JQ1, the most efficient drug inhibiting *MYCN* oncogene functions, does not show immunomodulatory capacity in terms of induction of ligands for NK cell-activating receptors and NK cell anti-tumor activity in NB cell lines.

## DISCUSSION

The antitumor activity of NK cells is controlled by the balance of inhibitory and activating signals. The expression of ligands for NK cell-activating receptors on tumor cell surface, that mediates activating signals, is crucial for an appropriate NK cell-mediated recognition and function [8]. The downmodulation of such ligands represent one of the mechanisms of immune-evasion for many tumors, including NB [4]. Anticancer drugs with immune-modulating effects can induce the immune system to attack cancer cells at a relatively low dose, thus contributing to better outcomes in cancer patients [38]. Several are the drug-mediated molecular mechanisms underlying the upregulation of activating ligands, representing efficient strategies aimed to boost the NK cell-based anti-tumor immunotherapy [9]. By contrast, drugs used in the clinical setting of NB did not show immune-modulatory effects [39], suggesting that further investigations are warranted to potentiate the NK cell-mediated immunotherapy of NB.

We recently demonstrated that the *MYCN* oncogene, which amplification represents the well-established predictor of poor prognosis, has an immunosuppressive role dampening the expression of ligands for NK cell-activating receptors [18]. Thus, *MYCN* targeting could be a therapeutic strategy to induce the expression of activating ligands and enhance NB cell susceptibility to NK cell-mediated immunotherapy. Among several mechanisms leading to the downregulation of MYCN expression/function, the BET bromodomain inhibitor JQ1 is a good candidate [26]. The use of JQ1 is very promising in several c-MYC-amplified tumors in which the downregulation of c-MYC leads to cell cycle arrest and apoptosis, thus inhibiting hypoxia and tumor progression [28, 30–33]. Moreover, JQ1 showed anti-angiogenesis activity contributing to arrest the growth of paediatric sarcoma [32]. JQ1 also dampens the expression of PD-L1, a major player involved in immune checkpoint mechanisms in NB [35] and in other cancers [40]. These data indicate that JQ1 treatment could represent a good strategy to cure NB allowing a better lymphocyte-mediated recognition of NB cells by reducing the PD1/PD-L1 immune checkpoint pathway established between lymphocytes and NB cells. Moreover, JQ1 increased the expression of MICA in multiple myeloma, since the downregulation of c-MYC leads to up-modulation of miR-125-5p and the consequent downregulation of its target gene IRF4, known as transcription repressor of MICA [41]. Differently to haematological malignancies, solid tumors, including NB, express lower levels of MICA and MICB and higher levels of ULBPs and ligands for DNAM-1 [4, 18, 42]. Moreover, the shedding of MICA has been associated with an immune escape strategy of NB [4]. Of note, the expression of miR-125-5p is very low in NB, correlates inversely with MYCN expression and is induced upon differentiation triggering [43]. This suggests that c-MYC/IRF4/miR-125b interplay could be less effective in NB compared with multiple myeloma in terms of MICA induction.

Herein, we explored the potential immune-modulatory effect of JQ1 on NB cell lines in terms of induction of ligands for NK cell-activating receptors. We treated NB cell lines at a pre-apoptotic dose of JQ1 for two main reasons. First, JQ1 is known to have an anti-tumor cytotoxic effect mainly in terms of growth arrest and apoptosis [30–32]. Second, the apoptosis, induced by drugs at cytotoxic doses, compromises the cell membrane integrity by down-regulating the expression of several ligands including those for NK cell-activating receptors [44]. The drug immune-modulatory effect in terms of induced surface molecules, such as ligands for NK cell-activating receptors, is generally experimentally explored at pre-apoptotic dose [9]. Moreover, in clinical setting, drug concentration varies in different body districts depending on the pharmacokinetic distribution and clearance mechanisms [45]. Thus, the cytotoxic function depends on drug concentration in specific tissue districts and could be less effective at low concentration. Of note, drugs that are cytotoxic at high concentration and have immune-modulatory effects, such as those to induce the expression of ligands for NK cell-activating receptors [9], at lower concentration, they represent one of the best molecular strategies to cure tumor patients.

Our data showed that the pre-apoptotic dose of JQ1, although efficiently downregulated the expression of MYCN, rendered NB cell lines more resistant to NK cells and probably also to T cells (not explored here), due to the shared expression of NKG2D on these lymphocytes. In spite of the cytotoxic activity against NB cells at higher doses, JQ1 is enable to upregulate the expression of activating ligands at pre-apoptotic dose, by impairing c-MYC levels, keeping impaired p53 function and downmodulating ROS levels. The consequent resistance of NB cells to immune NK cell-mediated attack should discourage the use of JQ1 in combination with the perspective NK cell-based immunotherapy of NB.

## MATERIALS AND METHODS

### Cell lines and reagents

Human NB cell lines were obtained as follow: SH-SY5Y and IMR-32 from the American Type Culture Collection (ATCC, Manassas, VA, USA), LA-N-5 from the Leibniz-Institut DSMZ (Deutsche Sammlung von Mikroorganismen und Zellkulturen GmbH, Braunschweig, Germany). NB cell lines were characterized by i) HLA class I typing by PCR-SSP sets (Genovision As, Oslo, Norway) according to the instructions of the manufacturer, and ii) array CGH [18, 39]. The human erythro-leukemia cell line K562 was purchased from ATCC and used as control target for NK cell functional assays. Cells were grown in RPMI 1640 medium supplemented with 10% FBS (Thermo Fisher Scientific, Waltham, MA, USA), 2 mM glutamine, 100 mg/ml penicillin and 50 mg/ml streptomycin (Euro Clone S.p.a., Milan, Italy). JQ1 was purchased from Selleckman and dissolved in DMSO at 1 mM.

### Antibodies, apoptosis, flow cytometry, Western blotting and ROS production

The following antibodies for flow cytometry were used: anti-CD107a-FITC (H4A3), anti-CD3-Alexa-700 (UCHT1), anti-CD56-PE-Cy7 (B159), anti-CD45-PE-Cy5 (HI30), purchased from BD Biosciences San Jose, CA, USA; anti-ULBP1-PE (170818), anti-ULBP2/5/6-PE (165903), anti-ULBP3-PE (166510), anti-CD155/PVR-PE (300907), anti-Nectin-2/CD112-APC (610603) purchased from R&D Systems Minneapolis, MN, USA. The following antibodies for Western blotting were used: anti-MYCN, anti-p53, anti-p21, anti-Actin (B8.4.B, FL-393, C-19 and I-19, respectively, Santa Cruz Biotechnology, Dallas, TX, USA), anti-c-MYC (Y69, OriGene, Rockville, MD, USA), and anti-MDM2 (2A10, Calbiochem-Millipore, Darmstadt, Germany). Apoptosis of tumor cells was evaluated with APC-conjugated AnnexinV (BD-Pharmingen) and propidium iodide (PI) (Sigma-Aldrich) and analyzed by flow cytometry. Flow cytometry was performed on FACSCantoII (BD Bioscences, San Jose, CA, USA) and analyzed by FlowJo Software. Whole-cell extracts were quantified by the bicinchoninic acid assay (Thermo Fisher Scientific, Waltham, MA, USA), resolved on 8-10% SDS-PAGE and electroblotted. Filters were probed with primary antibodies followed by goat anti-mouse IgG HRP conjugated (Jackson, West Grove, PA, USA).

ROS production was evaluated in NB cell lines, treated with DMSO or 0.5 μM of JQ1 for 48 hours, by using CellROX Deep Red Reagent (C10422, Invitrogen) and measured by flow cytometry.

### NK cell isolation

Human NK cells were isolated from peripheral blood mononuclear cells (PBMCs) of healthy donors with the RosetteSep NK-cell enrichment mixture method (StemCell Technologies, Vancouver, Canada) and Ficoll-Paque Plus (Lympholyte Cedarlane, Burlington, Ontario, Canada) centrifugation. NK cells were routinely checked for the CD3^-^CD56^+^ immunophenotype by flow cytometry and those with purity greater than 90% were cultured with 600 IU/mL of recombinant human IL-2 (PeproTech, Rochy Hill, NJ, USA) at 37°C and used up to 5 days after isolation.

### Degranulation and cytotoxicity assay

Degranulation assay was performed by co-culturing NK cells with target cells at 1:1 ratio for 3 hours, in complete medium in presence of anti-CD107a and in the last 2 hours of GolgiStop (BD Bioscence, San Jose, CA, USA). Then, cells were stained with anti-CD56 and anti-CD45 and expression of CD107a was evaluated by flow cytometry in the CD56^+^CD45^+^ subset. NK cell cytotoxic activity was tested by a standard 4-hour ^51^Cr-release assay. Specific lysis was converted to lytic units (L.U.) calculated from the curve of the percentage lysis. One lytic unit is defined as the number of NK cells required to produce 20% lysis of 10^6^ target cells during the 4 hours of incubation.

### Statistical analysis

Digital images of Western blots were analyzed by Image J (http://rsbweb.nih.gov/ij/index.html) and statistical significance of densitometric values was evaluated by the two-tailed unpaired Student's t-test. Normalized values were analyzed for correlation by the regression analysis using GraphPad software. *P* values not exceeding 0.05 were considered to be statistically significant.

## Abbreviations

NK cells: natural killer cells
BET bromodomain: bromo- and extra-terminal domain protein
JQ1: 2-(4-(4-chlorophenyl)-2,3,9-trimethyl-6H-thieno[3,2-f][1,2,4]triazolo[4,3-a][1,4]diazepin-6-yl)acetate), BET bromodomain-inhibitor
MYCN: v-Myc avian myelocytomatosis viral oncogene neuroblastoma derived homolog
c-MYC: v-Myc avian myelocytomatosis viral oncogene homolog
NKG2D: NK group 2 member D
DNAM-1: coactivating/adhesion DNAX-activating molecule 1
ULBPs: UL16-binding proteins
ROS: reactive oxygen species
